# Supplementary material for: Microglia Remodelling and Neuroinflammation Parallel Neuronal Hyperactivation Following Acute Organophosphate Poisoning
Source: Int J Mol Sci. 2022 Jul 26;23(15):8240. doi: 10.3390/ijms23158240 (PMC9332153; doi:10.3390/ijms23158240)
Supplement: Supplementary file 1 [file ijms-23-08240-s001.zip › Supplementary Materials explanation.pdf]

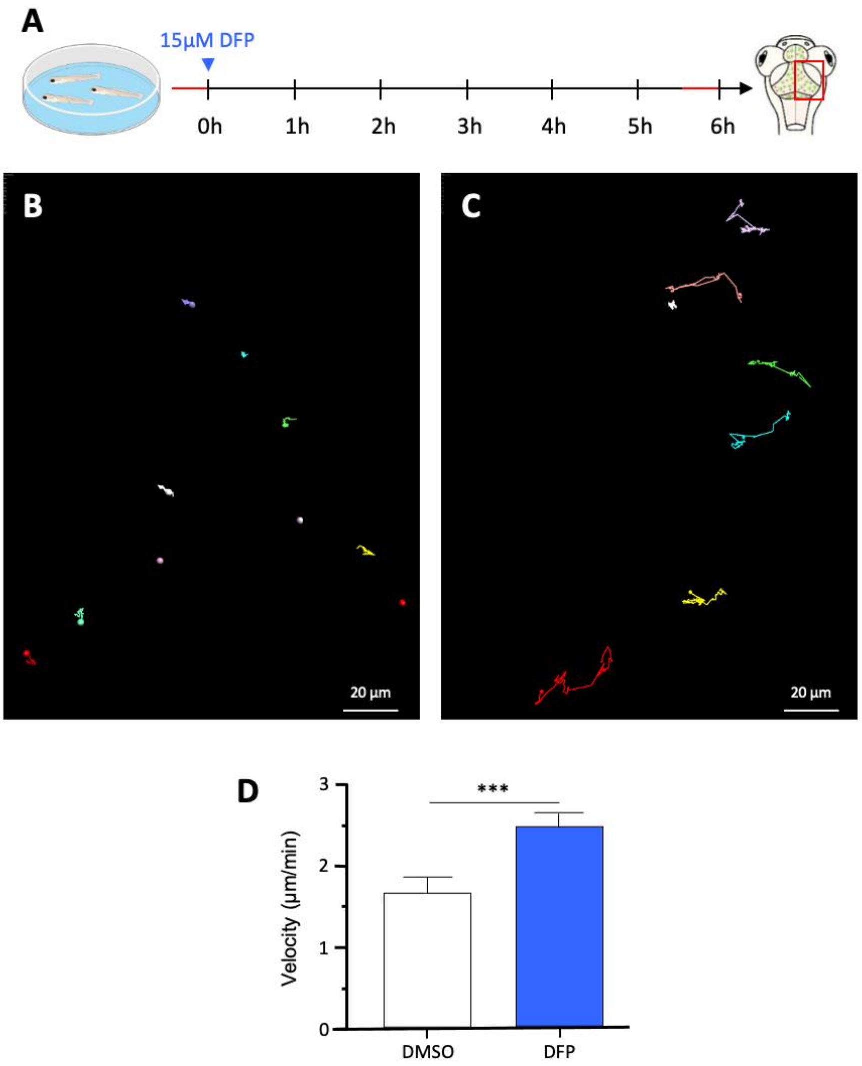

Supplementary Figure S1. DFP exposure increased the mobility of microglia

Timeline of treatment with red lines indicating the two 30-minute imaging periods. (**B-C**) Tracking of the cell body movements of microglia over a 30-minute period, before (**B**) and after 5.5 h of DFP exposure (**C**). The small coloured spheres correspond to the location of the cell body of individual cells at the start of the recordings. Scale bar represents 15 μm. (**D**) Mean distance travelled by microglial cells per minute, before and after DFP exposure ($N = 5$ larvae, $n = 48$ cells). Error bars on all graphs represent the standard error of the mean (SEM). Statistics: ***; $p < 0.001$.

Supplementary Table S1.

The primers (Eurofins Genomics, Ebersberg, Germany) used in this study.

il-1β	Forward	5′-CTT AAC CAG CTC TGA AAT GAT G-3′
	Reverse	5′-TGT CGC ATC TGT AGC TCA TTG-3′
il-8	Forward	5′-TGA CCA TCA TTG AAG GAA TGA G-3′
	Reverse	5′-CAT CAA GGT GGC AAT GAT CTC-3′
tnf-α	Forward	5′-TCA CGC TCC ATA AGA CCC AG-3′
	Reverse	5′-GAT GTG CAA AGA CAC CTG GC-3′
il-4	Forward	5′-GAG ACA GGA CAC TAC TCT AAG-3′
	Reverse	5′-GTT TCC AGT CCC GGT ATA TG-3′
il-10	Forward	5′-AAC GAG ATC CTG CAT TTC TAC-3′
	Reverse	5′-CCT CTT GCA TTT CAC CAT AT-3′
tgf-β3	Forward	5′-AAA ACG CCA GCA ACC TGT TC-3′
	Reverse	5′-CCT CAA CGT CCA TCC CTC TG-3′
tbp	Forward	5′-GTG CAC AGG AGC CAA AAG TG-3′
	Reverse	5′-GTT CAT AGC TGC TAA ACT GCT G-3′
c-fos	Forward	5′-AAC CAG ACT CAG GAG TTC AC-3′
	Reverse	5′-GGA GAA AGC TGT TCA GAT CTG-3′

Supplementary videos

Supplementary Video S1: Microglial dynamics in 5 dpf larva exposed to 1% DMSO

Time-lapse video of a dorsal view of the optic tectum of a 5 dpf larva exposed to 1% DMSO for 6 h. The video shows the last 45 minutes of the treatment, showing the dynamics of microglia, which mainly use their processes to monitor neighbouring cells. Time interval between frames: 40s. Video speed: 17 frames/s.

Supplementary Video S2: Microglial dynamics in 5 dpf larva exposed to DFP.

Time-lapse video of a dorsal view of the optic tectum of a 5 dpf larva exposed to 15 μM DFP for 6 h. The video shows the last 45 minutes of the treatment, showing the dynamics of microglia, which cell bodies appear to be more mobile than those of control larvae. Time interval between frames: 40s. Video speed: 17 frames/s.
