# Supplementary material for: Microglia Remodelling and Neuroinflammation Parallel Neuronal Hyperactivation Following Acute Organophosphate Poisoning
Source: Int J Mol Sci. 2022 Jul 26;23(15):8240. doi: 10.3390/ijms23158240 (PMC9332153; doi:10.3390/ijms23158240)
Supplement: Supplementary file 1 [file ijms-23-08240-s001.zip › Supplementary Table S1.pdf]

Supplementary Table S1.

The primers (Eurofins Genomics, Ebersberg, Germany) used in this study.

| | | |
|---|---|---|
| *il-1β* | Forward | 5’-CTT AAC CAG CTC TGA AAT GAT G-3’ |
| | Reverse | 5’-TGT CGC ATC TGT AGC TCA TTG-3’ |
| *il-8* | Forward | 5’-TGA CCA TCA TTG AAG GAA TGA G-3’ |
| | Reverse | 5’-CAT CAA GGT GGC AAT GAT CTC-3’ |
| *tnf-α* | Forward | 5’-TCA CGC TCC ATA AGA CCC AG-3’ |
| | Reverse | 5’-GAT GTG CAA AGA CAC CTG GC-3’ |
| *il-4* | Forward | 5’-GAG ACA GGA CAC TAC TCT AAG-3’ |
| | Reverse | 5’-GTT TCC AGT CCC GGT ATA TG-3’ |
| *il-10* | Forward | 5’-AAC GAG ATC CTG CAT TTC TAC-3’ |
| | Reverse | 5’-CCT CTT GCA TTT CAC CAT AT-3’ |
| *tgf-β3* | Forward | 5’-AAA ACG CCA GCA ACC TGT TC-3’ |
| | Reverse | 5’-CCT CAA CGT CCA TCC CTC TG-3’ |
| *tbp* | Forward | 5’-GTG CAC AGG AGC CAA AAG TG-3’ |
| | Reverse | 5’-GTT CAT AGC TGC TAA ACT GCT G-3’ |
| *c-fos* | Forward | 5’-AAC CAG ACT CAG GAG TTC AC-3’ |
| | Reverse | 5’-GGA GAA AGC TGT TCA GAT CTG-3’ |
