# Supplementary figures and images for: Microglia Remodelling and Neuroinflammation Parallel Neuronal Hyperactivation Following Acute Organophosphate Poisoning
Source: Int J Mol Sci. 2022 Jul 26;23(15):8240. doi: 10.3390/ijms23158240 (PMC9332153; doi:10.3390/ijms23158240)

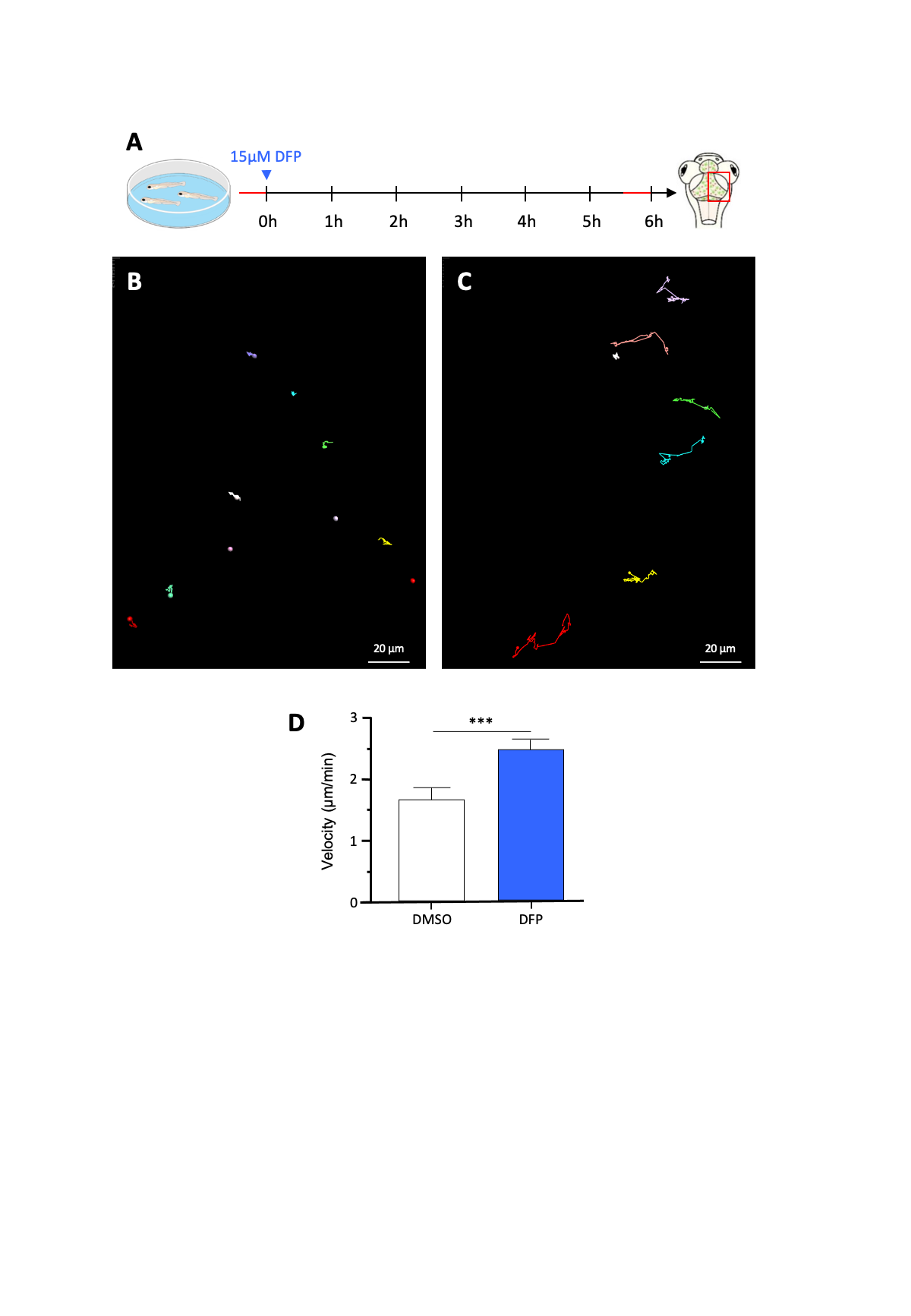

Supplement: Supplementary file 1 [file ijms-23-08240-s001.zip › Figure S1.tiff]
